# Expression of tumor pyruvate kinase M2 isoform in plasma and stool of patients with colorectal cancer or adenomatous polyps

**DOI:** 10.1186/s12876-020-01377-x

**Published:** 2020-07-29

**Authors:** Farideh Rigi, Aliakbar Jannatabad, Azra Izanloo, Reza Roshanravan, Hamid Reza Hashemian, Mohammad Amin Kerachian

**Affiliations:** 1 Razavi Cancer Research Center, Razavi Hospital, Imam Reza International University, Mashhad, Iran; 2 Department of Biotechnology, Faculty of Basic Science, Sabzevar Branch, Islamic Azad University, Sabzevar, Iran; 3 Medical Genetics Research Center, Mashhad University of Medical Sciences, Mashhad, Iran; 4 Department of Medical Genetics, Faculty of Medicine, Mashhad University of Medical Sciences, Mashhad, Iran; 5 Cancer Genetics Research Unit, Reza Radiotherapy and Oncology Center, Mashhad, Iran

**Keywords:** Colon, Malignancy, Blood, Non-invasive, M2-PK enzyme, diagnosis

## Abstract

**Background:**

Tumor pyruvate kinase M2 isoform (tM2-PK), which is an isoform of PK-glycolytic enzyme and appears on the surface of cancerous proliferating cells, has been used as a diagnostic biomarker for colorectal cancer (CRC). The aim of this study was to evaluate the tM2-PK measurement test for the diagnosis of CRCs and adenomatous polyps in plasma and stool samples in an Iranian population.

**Methods:**

In this prospective study, a total of 226 stool and 178 plasma samples were received from patients referred to colonoscopy units. tM2-PK enzyme was measured using two separate ScheBo-Biotech-AG ELISA kits for stool and plasma samples.

**Results:**

According to ROC curves, in the tumor group, at the cut-off value of 4 U/ml, the sensitivity of fecal tM2-PK test was 100% and the specificity was 68%, and in the polyp group, the sensitivity and specificity were 87 and 68%, respectively. For tumor detection in plasma specimens, a cut-off value > 25 U/ml has a sensitivity and specificity of 90.9 and 91.3%, respectively. Similarly, for polyp detection, a cut-off value > 19 U/ml has a sensitivity of 96.3% and the specificity of 85.5%.

**Conclusions:**

Based on our results, a cut-off range of 4.8–8 U/ml and >  8 U/ml could be used to detect polyp and tumor in stool samples, respectively. Similarly, a cut-off range of 19–25 U/ml and > 25 U/ml is recommended in plasma samples, suggesting tM2-PK test as a non-invasive assay to diagnose CRC and adenomatous polyps.

## Background

Colorectal cancer (CRC) is one of the leading causes of cancer morbidity and mortality worldwide [1, 2]. Its incidence rate has increased rapidly since it is associated with several risk factors related to lifestyle such as smoking, sedentary, obesity, alcohol abuse and diets containing high red and processed meats [3, 4]. Colonoscopy is currently claimed as the gold standard CRC screening tool [5, 6], however, it is expensive and may cause unexpected complications. Moreover, it is uncomfortable and painful for some patients to undergo colonoscopy examination. Thus, the compliance with colonoscopy for CRC screening is quite low [7]. Guaiac fecal occult blood test (gFOBT) is the most widely used noninvasive screening test for stool examination, although it has some limitations [8]. It is also inconvenient to perform since patients have to go on a restricted diet for several days prior to the test, which includes avoiding various types of food that may cause false peroxidase reaction and any antioxidants and non-steroidal anti-inflammatory drugs (NSAIDs) such as aspirin [9]. Another CRC screening test is the immunological fecal occult blood test (iFOBT) [10]. The low sensitivity of gFOBT and iFOBT may result in missing patients with CRC. Thus, a more effective screening tool is necessary [11, 12]. Based on methylation changes in stool and blood, two approved Food and Drug Administration (FDA) CRC detection kits respectively termed, ColoGaurd™ and Epi proColon® 2.0 CE kits are now available [13]. The relatively low sensitivity of these tests for early CRC and adenomatous polyp detection should be improved.

The majority of human tumors strongly overexpress M2 isoform of the glycolytic enzyme pyruvate kinase (M2-PK). This isoenzyme is released from tumor cells and is quantitatively detectable in body fluids. The measurement of tumor M2-PK has been proposed as a novel approach for early detection of CRC in the stool or blood of patients with CRC [10] since adenomas or CRC are usually associated with increased serum and stool levels of tumor M2-PK. Fecal M2-PK detects both bleeding and non-bleeding tumors as well as adenoma. It does not have false positive results originating from various noncancerous sources of bleeding, such as hemorrhoids and fissures. In contrast to FOBT, only one small stool sample (from a single stool passage) is requested without dietary restrictions for the test [14].

Hence, the aim of this study was to evaluate tumor M2-PK measurement test in plasma and stool samples to diagnosis CRC and adenomatous polyps in patients referred to colon clinics. Also, this study was performed to determine the best cut-off values for tumor M2-PK test in stool and plasma samples.

## Methods

### Patient population

In this prospective study, samples were taken in two separate centers including specialty hospital and oncology clinic of Mashhad, Iran. Participants were referred for colonoscopy because of positive screening, the presence of symptoms, or a positive family history. Participants admitted from April 2017 to June 2018, prior to colonoscopy handled their stool samples to the laboratory and at the same day, their blood samples were collected in EDTA tubes.

### Sample preparation

Two hundred and twenty-six stool and 178 plasma samples were taken from patients prior to colonoscopy. Sampling date was recorded. Minimum sample required for M2-PK test, was 100 mg of feces and 10 μL of plasma. Collected stool and plasma samples were kept frozen at − 20 °C prior to any experiments. Participants older than 30 years were categorized according to their age, sex, alcohol consumption, diabetes, smoking status and a family history of CRC. Patients with inflammatory bowel disease (Crohn and colitis disease) were not included in the study because recent reports indicate that the inflammatory bowel diseases can increase the M2-PK enzyme level [14–16].

Control group was defined as the participants with negative colonoscopy and case group was polyp (adenomatous)-positive or tumor-positive samples in colonoscopy examination. An expert gastrointestinal (GI) pathologist reported all pathology results. Patients suffering from solitary rectal cancer (1 case), hyperplastic (4 cases), retention (3 cases), inflammatory (2 cases) and mucosal (2 cases) polyps were excluded from the study since we targeted only adenomatous types of polyps. The histopathology report of one polyp resulted in unremarkable lesion which was also excluded. No patient with cancer also had polyps.

### M2-PK enzyme testing

Tumor M2-PK enzyme of samples was measured by two separate ScheBo-Biotech-AG ELISA kits (Giessen, Germany) for stool and plasma according to the manufacturer’s protocol. Based on colonoscopy and pathology results, participants were categorized as follow: among patients who their stool samples were collected 111 (49.1%) were normal, 76 (33.6%) patients had polyps, and 39 (17.3%) patients were suffering from CRC. In the plasma group, 69 (38.8%) were normal, 53 (29.7%) patients had polyps, and 56 (31.5%) patients were suffering from CRC. Only from 116 participants, both the stool and plasma samples were collected.

### Statistical analysis

The collected data were analyzed using SPSS version 19 and MedCalc statistical software. In addition to descriptive statistics, student's t-test, Pearson correlation testing, Chi square, ROC (receiver operating characteristics) curve, and ANOVA were used where applicable. A *p*-value < 0.05 was statistically significant in this study. Sensitivity and specificity expressed as ROC curve were calculated using colonoscopy results and histology as reference values.

## Results

In this study, 178 plasma samples were taken from patients including 96 men (53.9%) and 82 women (46.1%). The mean age of the patients whose stool and plasma samples were collected, was 54 and 57.22 years, respectively. Table 1 shows the number of normal, polyps and cancer patients in plasma and stool sample groups with the size of the polyps based on the colonoscopy reports. The lesions were located in rectosigmoid, ascending, descending, and transverse colon (Table 2). ANOVA test revealed no significant difference (*p* value < 0.05) in the location of tumor or polyp with a positive M2-PK test in either stool or plasma samples (Table 2).

**Table 1:** The number of normal, polyps and cancer patients in plasma and stool samples with the average size of the polyps based on the colonoscopy reports

Sample test	*Lesion type*	*Frequency*	*Percent*	*Polyp/Tumor size (cm)*	*< 1 cm, 1-2 cm, > 2 cm* *(%)*
*Stool*	Normal	111	49.1	–	–
	Cancer	39	17.3	3.0 (12.5)^a^	30.8–53.8-15.4
	Polyp	76	33.6	1.0 (4.0)^a^	57.6–16.9-25.5
	Total	226	100.0	–	–
*Plasma*	Normal	69	38.8	–	–
	Cancer	56	31.5	3.4 (14.0)^a^	23.2–64.3-12.5
	Polyp	53	29.7	1.5 (5.0)^a^	41.5–30.2-28.3
	Total	178	100.0	–	–

^a^Maximum size

**Table 2:** The location of tumor and polyps in stool and plasma samples

***Sample test***	***Lesion type***	***Ascending colon no. (%)***	***Transverse colon no. (%)***	***Descending colon no. (%)***	***Rectosigmoid no. (%)***	***p value***
	Tumor	12 (30.8%)	6 (15.4%)	0 (0%)	21 (53.8%)	0.288
*Stool*	Polyp	15 (19.7%)	7 (9.2%)	11 (14.5%)	43 (56.6%)	0.323
	Tumor	14 (25%)	6 (10.7%)	5 (8.9%)	31 (55.4%)	0.666
*Plasma*	Polyp	12 (22.6%)	6 (11.3%)	7 (13.2%)	28 (52.8%)	0.337

Neither the stool nor the plasma samples of tumor- and polyp-bearing patients showed significant differences between a positive M2-PK test result and the distribution of age, sex, diabetes, smoking and family history of tumor (*p* values > 0.05) except for tumor-bearing and normal subjects in terms of smoking with a positive M2-PK test (*p* value =0.011).

Although there was no significant difference between a M2-PK positive test and tumor (*p* value =0.967) or polyp (*p* value =0.074) size in stool samples, it was statistically significant in plasma samples (*p* values =0.0001 and = 0.005, respectively). The types of the adenomatous polyps were shown in Table 3.

**Table 3:** The types of the adenomatous polyps

***Sample***	***Type of polyp***	***Tubular adenoma***	***Tubulovillous adenoma***	***Villous adenoma***	***Sessile serrated adenoma***	***Total***
*Stool*	*Multiple adenomatous*	13 (17.1%)	8 (10.52%)	1 (1.31%)	2 (2.63%)	24 (31.57%)
	*Single adenomatous*	43 (56.57%)	8 (10.52%)	0	1 (1.31%)	52 (68.42%)
*Plasma*	*Multiple adenomatous*	9 (16.98%)	4 (7.54%)	2 (3.77%)	2 (3.77%)	17 (32.07%)
	*Single adenomatous*	26 (49.05%)	8 (15.09%)	0	2 (3.77%)	36 (67.92%)

**Fig. 1:**
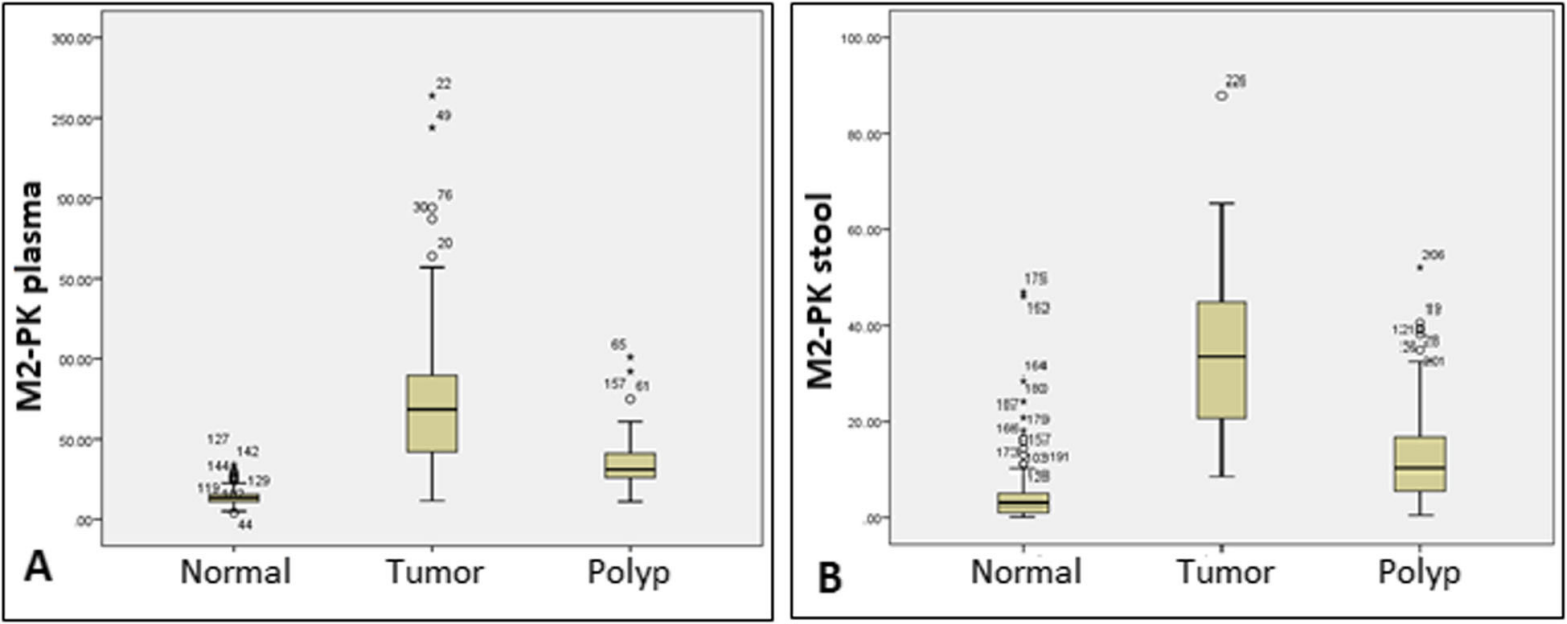
Comparison of M2PK plasma and stool test results in 3 groups (normal, polyp, tumor)

ANOVA test was used to compare the difference between the results of M2-PK stool and plasma samples in the three groups of normal, patients with polyp, and patients with adenocarcinoma, indicating significant differences between the groups (both tests had p values =0.0001) (Fig. 1). Besides, Chi-square test was used to compare the levels of M2-PK in tumor-/ polyp-bearing patients with controls in stool and plasma samples (Table 4).

**Table 4:** The levels of M2-PK in tumor-/ polyp-bearing patients with controls in stool and plasma samples

***Sample type***	***Lesion type***	***Chi-squared p value***	***Sensitivity***	***Specificity***	***Positive Predictive value***	***Negative predictive value***
***Cut-off value of 4 U/ml***						
*Stool*	Tumor	0.0001	100%	68%	52.7%	100%
	Control					
	Polyp	0.0001	87%	68%	65%	88%
	Control					
***Cut-off value of 15 U/ml***						
*Plasma*	Tumor	0.0001	98%	74%	75%	98%
	Control					
	Polyp	0.0001	98%	74%	74%	98%
	Control					

**Fig. 2:**
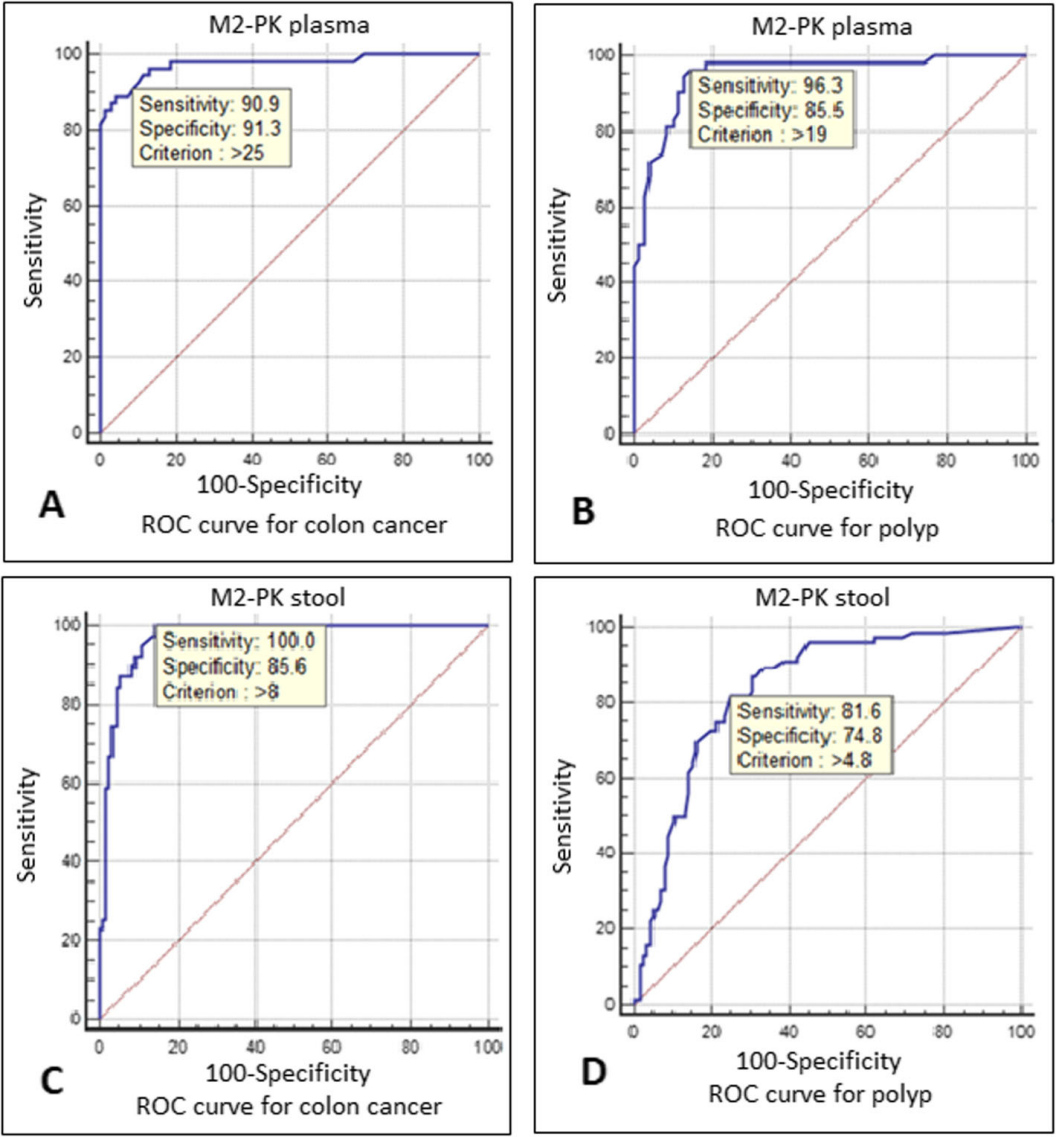
Receiver operating characteristic (ROC) curve for tumor/polyp M2PK plasma and stool

In the current study, we used ROC curves to determine the best cut-off value for tumor/polyp M2-PK test (Table 5 and Fig. 2). For tumor detection in plasma specimens, a cut-off value > 25 U/ml has a sensitivity and specificity of 90.9 and 91.3%, respectively (Fig. 2a). Similarly, for polyp detection, a cut-off value > 19 U/ml has a sensitivity of 96.3% and the specificity of 85.5% (Fig. 2b). The AUC of polyp and tumor data is 0.95 and 0.975 respectively, which reveals that the overall discriminatory power of the test is quite high. Also for tumor detection in stool specimens, with a cut-off value > 8 U/ml the test sensitivity is 100% and the specificity is 85.6% (Fig. 2c). For polyp detection, a cut-off value > 4.8 U/ml has a sensitivity and specificity of 81.6 and 74.8%, respectively (Fig. 2d). AUC of polyp data is 0.834 and of tumor data is 0.969, which indicates that the overall discriminatory power of the test is high.

**Table 5:** The cut-off values based on ROC curves for tumor/polyp M2-PK test in stool and plasma samples

***Sample type***	***Lesion type***	***Suggested cut-off value based on ROC (U/ml)***	***Sensitivity***	***Specificity***	***AUC***
*Stool*	Tumor	> 8	100%	85.6%	0.969
	Polyp	> 4.8	81.6%	74.8%	0.834
*Plasma*	Tumor	> 25	90.9%	91.3%	0.975
	Polyp	> 19	96.3%	85.5%	0.95

## Discussion

In the current study, our stool and plasma study did not show a significant difference between a positive M2-PK test result and the distribution of age, sex, diabetes and family history of tumor in tumor- or polyp-bearing patients. There was only a significant difference between the results of M2-PK test in plasma samples of tumor-bearing subjects and normal subjects in terms of smoking (*p* value = 0.011), although it was not seen in polyp-bearing subjects.

These findings were in consistent with the findings of U Haug et al., which reported that the subgroup of the ESTHER study did not differ from the whole ESTHER study population with respect to the distribution of age, sex, body mass index, smoking status and a family history of CRC. However, current smokers showed more frequently increased levels of tumor M2-PK in stool compared to never and former smokers (*p* value = 0.003) [17]. In a similar study, male and female groups showed no significant differences in age or fecal tumor M2-PK levels although a highly significant difference was found between the tumor M2-PK level for participants aged 20–49 years (median M2-PK of 0.66) and 50–79 years (median M2-PK of 0.086) [18]. Furthermore, in another study with 1082 participants (mean age 63 years, 50% females) the median tumor M2-PK level in the whole study population was 1.3 U/ml (0.3–3.3). Median tumor M2-PK levels did not alter by gender, but tended to be higher in older age groups (*p* value = 0.002). In addition, the sensitivity and specificity did not vary by sex of stool samples. The specificity tended to be lower in older age groups (*p* value = 0.001) but the sensitivity did not vary by age [19]. They have also showed that the average serum M2-PK value among 158 normal individuals was 2.96 U/mL, which was not affected by gender or age [20]. The study of Mohamed El–Tantawy Ibrahim and his colleagues revealed that there was no significant difference between patients with colon cancer and control groups considering the age and sex. Moreover, 32% of their patients were smokers compared to only 3.3% of the control group, which was statistically significant (*p* value < 0.05) [3].

In our study, although in M2-PK plasma experiment the size of the tumor or polyp was statistically different in the tumor- or polyp-bearing patients in compare to controls, there was no difference between these groups in the M2-PK stool experiment. This was consistent with the study of Yogesh M. et al. which reported that in patients undergoing colonoscopy 31 had adenomatous polyps, 21 had small adenomas (< 10 mm) and 10 had large adenomas (> = 10 mm). Median stool M2-PK in the small and large adenoma groups was 2.9 U/ml and 1.5 U/ml respectively, which was not statistically significant when compared with normal groups. M2-PK was reported positive in 25.8% of adenomas regardless of their sizes; however, FOBT seemed to be more associated with the size of the lesion [11]. In addition, in a similar study with 50 patients suffering from an adenomatous disease, 22 were found to have a single polyp greater than 1 cm in size. There was no significant difference in the M2-PK concentration detectable in the feces of patients with polyps less or above 1 or even the size of 5 cm [21].

In our study, ANOVA test revealed no significant difference in the location of tumor or polyp with a positive M2-PK test in either stool or plasma samples. However, Haug et al. showed that there was a statistically difference (*p* value < 0.001) in tumor M2-PK levels in stool of ESTHER participants based on the location of the tumor. In their study with the cut-off value of 4 U/ml, overall sensitivity was 68% with a clear difference between colon cancer (85%) and rectum cancer (56%) [17]. In our results, at the cut-off value of 4 U/ml, the test sensitivity for the stool samples of polyp-bearing groups was 87%, specificity was 68%, PPV was 65% and NPV was 88%. The sensitivity of fecal M2-PK test was higher in tumor-bearing group (100%) than in polyp-bearing group (87%). In addition, NPV was 100% in tumor-bearing group, meaning that if the level of fecal M2-PK of an individual is determined less than 4 U/ml, the probability for a tumor is almost zero. In contrast, regarding the low PPV of M2-PK test for detecting tumor and polyp in stool, any result higher than 4 U/ml can be false positive indicating a low specificity of the test. In a study performed by Kumar et al., fecal tumor M2-PK had a sensitivity of 73–92% at a cut-off value of 4 U/ml in compared to 50% sensitivity for Guaiac fecal test. They also indicated that, at a diagnostic cut-off value of 15 U/ml for plasma tumor M2-pyruvat kinase, sensitivity, specificity, PPV and NPV were 57.3, 89, 85.7 and 64.8%, respectively [22]. Based on our results, with the same cut-off value for plasma tumor M2-PK, the sensitivity, specificity, PPV and NPV were 98, 74, 75 and 98%, respectively. In a multi-center study on 317 subjects with a cut-off value of 4 U/ml, fecal M2-PK assay had a sensitivity, specificity, PPV and NPV of 81.1, 71.1, 61.9, and 86.7% respectively to detect CRC [23]. Also, in another study with 328 patients and the tumor M2-PK cut-off level of 4 U/mL, the sensitivity, specificity, PPV, and NPV were 71.4, 71.0, 73.5, and 94.4%, respectively [20]. In a study by Hisham K. Dabbous et al., M2-PK was the most sensitive and specific test in differentiating CRC from control subjects in fecal samples with sensitivity and specificity of 75, and 100%, respectively [14].

In the current study, in order to achieve the best performance of tumor/polyp M2-PK measurement test in stool and plasma samples different cut-offs have been evaluated.

## Conclusions

A cut-off range of 4.8–8 U/ml in stool samples can detect polyp and a cut-off value > 8 U/ml can detect tumor. In addition, a cut-off range of 19–25 in plasma samples can detect polyp and a cut-off value > 25 can detect tumor. The relatively high specificity and sensitivity of tumor M2-PK measurement test in stool and plasma samples of patients with CRC and polyp indicate that this test has the potential be used as a non-invasive diagnostic tool in CRC and colon adenomas detection although for general screening, a study on a general population with larger sample sizes should be performed in advance.

## Data Availability

All data are included in this published article. Any additional information related to this study is available from the author for correspondence upon reasonable request.

## Abbreviations

gFOBT: Guaiac fecal occult blood test
tM2-PK: Tumor pyruvate kinase M2 isoform
CRC: Colorectal cancer
NSAIDs: Non-steroidal anti-inflammatory drugs
iFOBT: Immunological fecal occult blood test
FDA: Food and Drug Administration
ROC: Receiver operating characteristics
AUC: Area under the cure
PPV: Positive predictive value
NPV: Negative predictive value
